# Causal Association of Chronic Venous Insufficiency and Cardiovascular Diseases: A Univariable and Multivariable Mendelian Randomization Study

**DOI:** 10.31083/j.rcm2510357

**Published:** 2024-10-08

**Authors:** Xiaobo Guo, Kui Zhang, Yiping Sun, Ran Dong

**Affiliations:** ^1^Department of Cardiac Surgery, Beijing Anzhen Hospital, Capital Medical University, 100029 Beijing, China

**Keywords:** chronic venous insufficiency, cardiovascular diseases, Mendelian randomization, heart failure, atrial fibrillation

## Abstract

**Background:**

The causal relationship between chronic venous insufficiency (CVI) and cardiovascular diseases (CVDs) has yet to be elucidated. Herein, we implement Mendelian randomization (MR) analysis to investigate the causal association.

**Methods:**

A two-sample MR approach using genetic data from FinnGen and genome-wide association studies (GWAS) Catalog was applied to investigate the causal relationship between CVI and CVDs. This study assessed 77 single nucleotide polymorphisms (SNPs) as instrumental variables, employing random-effect inverse-variance-weighted MR, weighted median, Egger regression, Mendelian Randomization Pleiotropy RESidual Sum and Outlier (MR-PRESSO), and Robust Adjusted Profile Score (RAPS) methods. Multivariable MR (MVMR) considered confounding factors.

**Results:**

Genetically predicted CVI was associated with reduced heart failure risk (odds ratio (OR) = 0.96, 95% confidence interval (95% CI): 0.93–0.99, *p* = 0.025) and increased atrial fibrillation risk (OR = 1.06, 95% CI: 1.03–1.09, *p* = 0.0002). MVMR, adjusting for venous thromboembolism (VTE), lower limb ulceration, obesity, smoking, and alcohol, attenuated these associations. No significant links were found with hypertension, aortic aneurysm, coronary artery disease, myocardial infarction, valvular heart disease, or stroke.

**Conclusions:**

This MR study supports an association between CVI and CVDs, which may imply CVI should be monitored during the treatment of heart failure and atrial fibrillation.

## 1. Introduction

Although notable progress has been made recently in managing cardiovascular diseases (CVDs), it continues to be the foremost cause of morbidity and mortality worldwide. These persistent challenges underscore the intricate nature of CVDs, necessitating a multifaceted approach to address its complexity. However, further exploration and refinement of preventive strategies and treatment modalities are imperative to alleviate the burden of CVDs on individuals and healthcare systems. Furthermore, with established risk factors such as obesity and smoking, CVDs have been identified in association with numerous other diseases [1, 2].

Chronic venous insufficiency (CVI) describes a common condition that affects the venous system of the lower extremities, with persistent ambulatory venous hypertension causing various pathologies, including pain, edema, skin changes, and ulcerations [3]. The prevalence of CVI has been reported to vary between 6.6% and 40.8% [3, 4]. As CVI progresses, the occurrence of venous thromboembolism (VTE) or lower extremity venous ulcers results in significant pain and economic burden. Although traditionally considered two independent pathological changes, increasing evidence suggests a correlation between CVI and CVDs. Firstly, they share common risk factors [1, 5]. Secondly, both CVI and CVDs manifest similar disease characteristics: inflammation and thrombosis resulting from venous or arterial endothelial dysfunction [6, 7, 8]. A 2021 population study established an association between CVI and CVDs, suggesting that CVI may exacerbate the risk of mortality associated with CVDs [4]. Subsequently, the proposition that “the legs are a pathway to the heart” garnered significant attention [9]. Therefore, numerous findings include shared risk factors and a tight connection of physiopathological roles for vessels in CVI and CVDs; thus, comprehensive characterization of the causal relationship between CVI and CVDs could shed light on more promising therapeutic strategies in clinical practice.

The relationship between CVI and CVDs may be explained by two potential scenarios: (1) shared etiology or risk factors and (2) the possibility of a causal effect. While conducting randomized controlled trials to establish the causal relationship between these two diseases is challenging, Mendelian randomization (MR) represents an alternative robust method. MR employs genetic variations as surrogate variables or “instrumental variables” to simulate randomized controlled trials (RCTs), aiming to determine proposed risk factors. Therefore, this study employed a two-sample MR approach to investigate the causal relationship between CVI and CVDs.

## 2. Methods

### 2.1 Study Design and Data Sources

We evaluated the causal effects between CVI and CVDs utilizing a two-sample MR approach. Our MR design rationale was grounded on three assumptions: (1) the genetic variants exhibit robust associations with CVI; (2) the genetic variants lack associations with other confounding factors; (3) the genetic variants are exclusively associated with the clinical outcome through CVI.

The single nucleotide polymorphisms (SNPs) linked with CVI were acquired from the FinnGen consortium (R10), comprising 2060 cases and 357,111 controls. The SNPs associated with CVDs were sourced from the genome-wide association studies (GWAS) Catalog, encompassing heart failure (n = 977,323), essential hypertension (n = 456,348), stroke (n = 446,696), atrial fibrillation (AF, n = 1,030,836), coronary atherosclerosis (n = 456,348), myocardial infarction (n = 58,825), aortic aneurysm (n = 456,348), and cardiac valvular disease (n = 484,598). The FinnGen consortium (R10) provided the SNPs related to risk factors, which include VTE (n = 41,281), lower limb ulcers (n = 389,610), obesity (n = 412,055), smoking (n = 155,619), and alcohol use disorders (n = 412,181). Comprehensive source information regarding the data utilized in the present study is delineated in Table 1. Ethics approval was unnecessary for this study since all used GWAS statistics were publicly available and previously approved by respective ethical review boards.

**Table 1.** **Description of of data sources for analyses**.

Traits	GWAS ID	Sample size	Cases	Controls	Ancestry	PubMed ID
Outcomes						
Essential hypertension	GCST90043949	456,348	1105	455,243	European	34737426
Aortic aneurysm	GCST90044010	456,348	258	456,090	European	34737426
Coronary atherosclerosis	GCST90043957	456,348	16,041	440,307	European	34737426
Myocardial infraction	GCST011365	58,825	14,825	44,000	European and unknown	33532862
Heart failure	GCST009541	977,323	47,309	930,014	European	31919418
Arial fibrillation	GCST006414	1,030,836	60,620	970,216	European	30061737
Cardiac valvular disease	GCST90038612	484,598	3742	480,856	European	33959723
Stroke	GCST006906	446,696	40,585	406,111	European	29531354
Exposures						
CVI	finngen_R10_I9_VARICVESOTH	359,171	2060	357,111	European	
VTE	finngen_R10_I9_VTE	412,181	21,021	391,160	European	
Lower limb ulcer	finngen_R10_L12_ULCERLOWLIMB	389,610	4101	385,509	European	
Obesity	finngen_R10_E4_OBESITY	412,055	23,971	388,084	European	
Smoking	finngen_R10_SMOKING	155,619	3778	151,841	European	
Alcohol use disorder	finngen_R10_AUD_SWEDISH	412,181	24,070	388,111	European	

CVI, chronic venous insufficiency; VTE, venous thromboembolism; GWAS ID, genome-wide association studies identification.

### 2.2 Selection of Genetic Instruments

We discerned independent SNPs linked with CVI by applying three distinct criteria. Firstly, SNPs were chosen based on a genome-wide significance threshold of *p* < 5 × 10^-8^. Then, we assessed the independence between selected SNPs through paired linkage disequilibrium analysis. SNPs were clumped and discarded at linkage disequilibrium (LD) r^2^ > 0.001. To exclude weak instrumental variables, SNPs with F-statistic <10 were removed. Secondly, each instrumental variable was examined in PhenoScanner to identify any prior associations with potential confounding factors, including high blood cholesterol, high blood pressure, smoking, overweight, obesity, diabetes, metabolic syndrome, kidney disease, insomnia, obstructive sleep apnea (OSA), alcohol use, physical activity, and height. Finally, we identified 77 SNPs as instrumental variables.

### 2.3 Univariable MR

The primary analysis employed the random-effect inverse-variance-weighted (IVW)-MR method. Additionally, we conducted sensitivity analyses using six complementary methods, including the weighted median, MR-Egger regression, Simple mode, Robust Adjusted Profile Score (RAPS), weighted mode, and Mendelian Randomization Pleiotropy RESidual Sum and Outlier (MR-PRESSO). The directionality test was conducted to identify any bias from reverse causality in the observed association. Lastly, we conducted a leave-one-out analysis to assess whether any single SNP significantly influenced the overall IVW method.

### 2.4 Multivariable MR

Adjustments were implemented in the multivariable Mendelian randomization (MVMR) analysis to account for CVI-related complications and CVD risk factors. In the clinical context, heart failure is influenced by various factors, and CVI-associated VTE can exacerbate heart failure, while the inflammatory state caused by CVI-related lower extremity venous ulcers can also worsen heart failure. Based on the univariable MR analysis and the multifunctional screening process of instrumental variables using PhenoScanner, we identified obesity, smoking, and alcohol as risk factors for atrial fibrillation.

Similar to the univariable Mendelian randomization (UVMR), instrumental variables must simultaneously meet the criteria of being genome-wide significant (*p* < 5 × 10^-8^), LD r^2^ > 0.001, and F-statistics >10 for MVMR. The analysis utilizes the IVW, weighted median, Egger regression, and Lasso regression methodologies. The heterogeneity of the IVW method is appraised through the Q-statistic, while pleiotropy is assessed using Egger regression. Priority was given to the Lasso regression over the IVW model in the presence of heterogeneity [9].

### 2.5 Statistical Analysis

The effect estimates of genetically predicted CVI on CVDs were presented as odds ratios (ORs) and their corresponding 95% confidence intervals (95% CIs). Bonferroni correction was used to reduce false positives from multiple tests, setting the significance threshold at 0.006 (0.05/8). Here, *p*-values between 0.05 and 0.006 were considered suggestive evidence of potential causal associations. The analyses conducted in this study were executed using R software (version 4.3.2, R Foundation for Statistical Computing, Vienna, Austria), employing the R packages “TwoSampleMR” (version: 0.5.8), “MRPRESSO” (version: 1.0), “MendelianRandomization” (version 0.9.0), and “MVMR” (version 0.4).

## 3. Results

We identified 120 SNPs significantly associated with CVI from the GWAS study (*p* < 5 × 10^-8^) after removing variants in linkage disequilibrium (r^2^ < 0.001, 10,000 kb). Then, 43 SNPs related to CVDs and risk factors were excluded according to the PhenoScanner database. Only variants available for outcome traits were used, and proxy SNPs were not applied. All selected SNPs demonstrated F-statistics >10 (ranging from 391 to 9408), indicating the absence of weak instruments. Finally, 77 SNPs were employed for MR analysis, and any outliers identified using the MR-PRESSO method were excluded.

### 3.1 UVMR Analysis

**Fig. 1.**
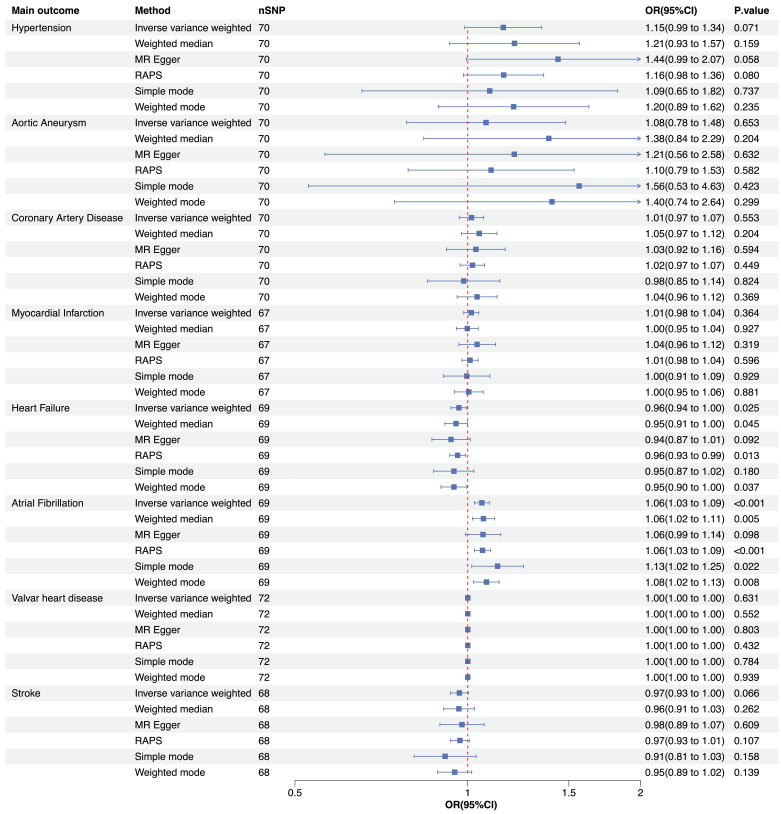
**Forest plot illustrating the UVMR analysis investigating the impact of CVI on CVDs**. UVMR, univariable Mendelian randomization; CVI, chronic venous insufficiency; CVD, cardiovascular disease; nSNP, number of single nucleotide polymorphisms; OR, odds ratio; 95% CI, 95% confidence interval; MR, Mendelian randomization; RAPS, Robust Adjusted Profile Score.

**Fig. 2.**
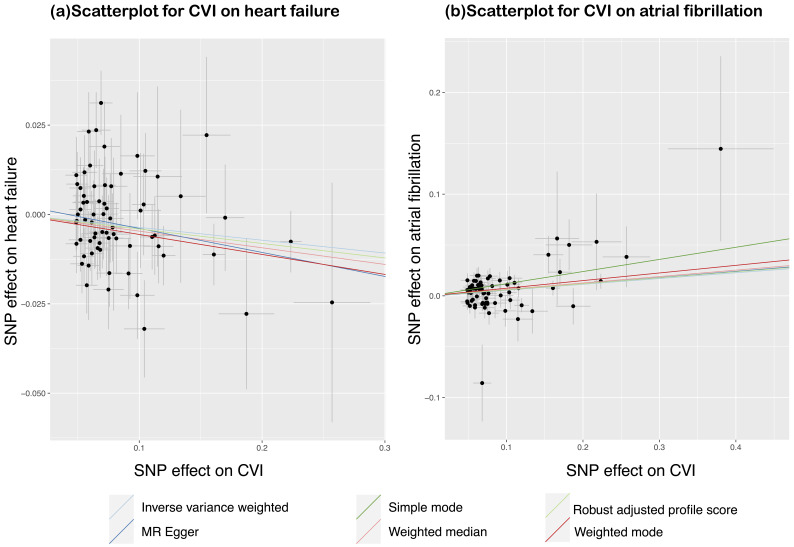
**Scatter plot of UVMR estimates of genetic risk for CVI affecting heart failure and atrial fibrillation**. The genetic effects of CVI against their effects on heart failure (a) and atrial fibrillation (b), alongside horizontal and vertical lines indicating the corresponding standard errors. The slope of each line represents the estimated genetic effect obtained from different Mendelian randomization (MR) methods. UVMR, univariable Mendelian randomization; CVI, chronic venous insufficiency; SNP, single nucleotide polymorphism.

After outliers identified using the MR-PRESSO method were deleted, we examined the association of CVI with various cardiovascular conditions, including hypertension, aortic aneurysm, coronary artery disease, myocardial infarction, heart failure, atrial fibrillation, valvar heart disease, and stroke. The IVW method was employed as the primary analytical approach. The possible associations between CVI and CVDs were identified (Fig. 1). The IVW method revealed that CVI was potentially associated with heart failure (OR = 0.96, 95% CI: 0.93–0.99, *p* = 0.025) and strongly associated with atrial fibrillation (OR = 1.06, 95% CI: 1.03–1.09, *p* = 0.0002). Various methods, including weighted median, MR Egger, MR-RAPS, simple mode, and weighted mode, were adopted, and the results were highly consistent with the IVW results, reflecting a tight association between CVI and CVDs (Figs. 1,2). Nevertheless, no significant associations were found between CVI and hypertension, aortic aneurysm, coronary artery disease, myocardial infarction, valvar heart disease, or stroke.

**Fig. 3.**
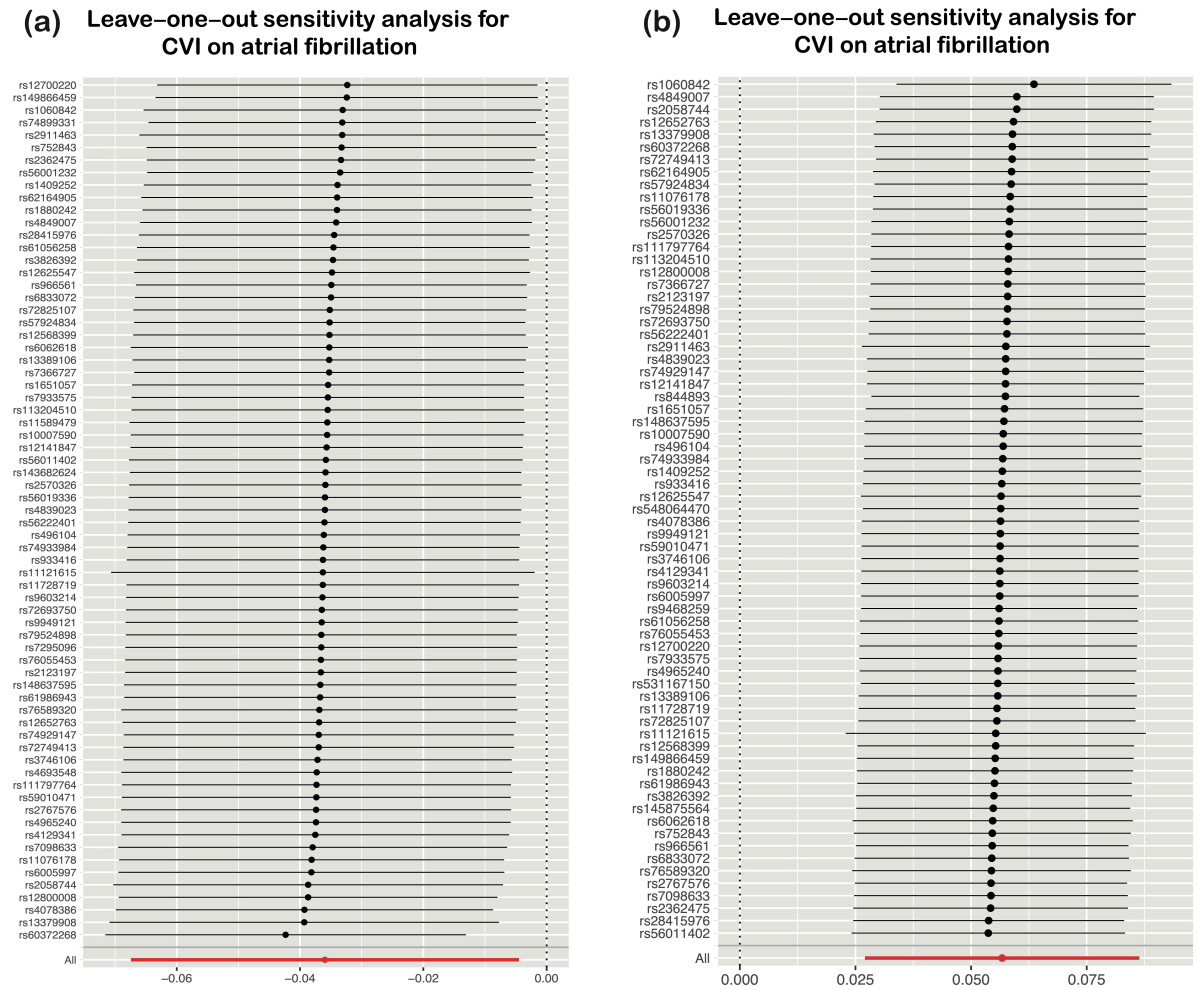
**Leave-one-out analysis of UVMR estimates the genetic risk of CVI on heart failure and atrial fibrillation**. Each black box represents the odds ratio (OR) for individual single nucleotide polymorphisms (SNPs) as determined by the inverse variance weighted (IVW) method after leaving the corresponding SNP in turns. The black box labeled ‘ALL’ represents the pooled IVW MR estimate. The horizontal lines indicate the 95% confidence intervals (CI). UVMR, univariable Mendelian randomization; CVI, chronic venous insufficiency.

For sensitivity analyses, the MR-Egger intercept suggested the absence of directional pleiotropy. Cochran’s Q statistics indicated that heterogeneity might exist for a causal association between CVI and atrial fibrillation (*p* = 0.02), while there was no heterogeneity between CVI and heart failure (*p* = 0.16). Steiger filtering revealed no SNPs with reverse causation, affirming the reliability of the causal direction (Table 2). In addition, the leave-one-out method showed that no single SNP was driving a potential causal correlation between CVI and heart failure and atrial fibrillation (Fig. 3).

**Table 2.** **Sensitivity analyses**.

Main outcome	Pleiotropy test		Heterogeneity test				Directionality test
	MR-Egger		MR-Egger		IVW		Steiger
	Intercept	*p*	Q	Q_*p*val	Q	Q_*p*val	*p* value
Hypertension	–0.0202	0.20	63.892	0.62	65.56	0.60	
Aortic aneurysm	–0.0104	0.75	62.752	0.66	62.86	0.69	
Coronary artery disease	–0.0016	0.75	96.007	0.02	94.15	0.02	
Myocardial infarction	–0.0022	0.49	74.581	0.19	75.14	0.21	
Heart failure	0.0028	0.39	78.799	0.15	79.69	0.16	5.30 × 10^–⁢60^
Atrial fibrillation	–0.0004	0.89	93.635	0.02	93.66	0.02	1.14 × 10^–⁢164^
Valvar heart disease	<0.0001	0.63	52.408	0.94	52.64	0.95	
Stroke	–0.0009	0.81	68.158	0.44	68.22	0.44	

MR, Mendelian randomization; IVW, inverse-variance-weighted.

### 3.2 MVMR Analysis

Due to heterogeneity in the multivariable Mendelian randomization (MVMR) analysis, the Lasso regression approach was employed to assess the causal effect of genetic susceptibility to CVI on heart failure and atrial fibrillation.

**Fig. 4.**
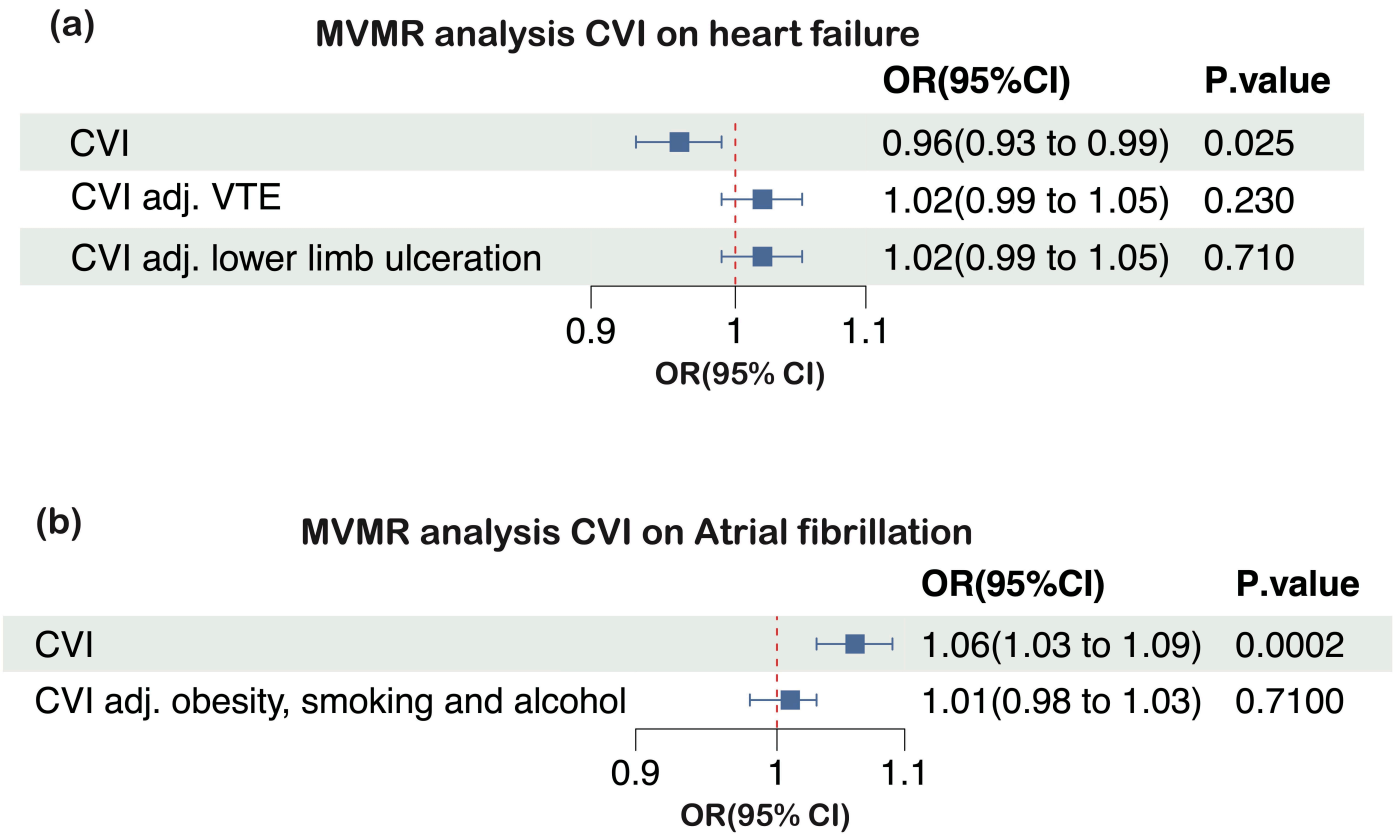
**Multivariable MR (MVMR) estimates for the effects of CVI on (a) heart failure and (b) atrial fibrillation, before and after accounting for putative mediating factors**. CVI, chronic venous insufficiency; MR, Mendelian randomization; OR, odds ratio; 95% CI, 95% confidence interval; VTE, venous thromboembolism.

To investigate the association between CVI and the risk of heart failure, potential mediation was explored, including VTE and lower limb ulceration. These factors are not only linked to CVI but are also recognized as associations with heart failure. In the MVMR analysis, the causal effect of CVI on heart failure was significantly attenuated after adjustment for VTE (OR = 1.02, 95% CI: 0.99–1.05, *p* = 0.23; conditional F-statistic for CVI = 26), and lower limb ulceration (OR = 1.02, 95% CI: 0.99–1.05, *p* = 0.26; conditional F-statistic for CVI = 16) (Fig. 4a).

Obesity, smoking, and alcohol consumption are common risk factors for both CVI and atrial fibrillation. After adjusting for these factors, no significant associations were observed between CVI and AF (OR = 1.05, 95% CI: 0.98–1.03, *p* = 0.71; conditional F-statistic for CVI = 21) (Fig. 4b).

## 4. Discussion 

This study represents the first two-sample MR analysis aiming to comprehensively elucidate the causal relationship between CVI and CVDs utilizing GWAS data. Our results suggest that a genetic predisposition to CVI is correlated with a potentially reduced risk of heart failure and a significantly increased risk of atrial fibrillation. However, upon adjustment for confounding factors through MVMR, this causal relationship becomes non-significant. Furthermore, there is limited MR evidence supporting a potential causal effect between CVI and hypertension, aortic aneurysm, coronary artery disease, myocardial infarction, valvular heart disease, and stroke risk.

### 4.1 CVI and Heart Failure

Our MR results indicate that CVI serves as a protective factor against heart failure. This contrasts with previous research findings, as previous studies suggest that CVI aggravates heart failure [4, 10]. One potential explanation is that during the early stages of heart failure, the venous vasculature, serving as a capacitance vessel, undergoes dilation, alleviating the cardiac preload. In cases of venous insufficiency, the capacity of lower extremity veins increases more noticeably. During this period, CVI may act as a protective factor, preventing premature cardiac decompensation. However, as CVI progresses, complications such as CVI-induced VTE, lower extremity venous ulcers, or bleeding from anticoagulant therapy for VTE may exacerbate heart failure-related mortality. The previous study employed COX regression analysis [4, 10], which assumes proportional hazards, meaning the effect of risk factors is constant over time. Given the varying impact of CVI on heart failure at different stages of the disease, the two methods can yield divergent conclusions.

VTE and venous leg ulcers (VLUs) represent late complications of CVI, concurrently influencing CVDs [11, 12]. Thrombotic events in heart failure patients may be more prevalent than anticipated, with autopsy studies revealing thrombotic event-related deaths accounting for 42.2% of total deaths in heart failure patients, with pulmonary embolism comprising 36.1% [13]. While there is limited research on the relevant mechanisms, theoretical considerations propose that endothelial dysfunction in the venous system may influence venous tension, leading to alterations in venous capacitance and compliance, subsequently impacting the development of heart failure.

A population-based cohort study found that individuals at CVI grade 3 had a 1.83 times increased risk of mortality and a 2.04 to 2.06 times increased risk of heart failure, acute coronary syndrome, and ischemic stroke compared to matched controls, while such associations were not observed in patients with grades 1–2 [14]. VLU in patients with chronic venous insufficiency has been strongly correlated with atrial fibrillation and right heart failure [11]. Collectively, this evidence indicates that the impact of CVI on CVDs changes as CVI progresses. However, the MR study did not support a bidirectional causal connection between VLU and heart failure [15].

In our study, including VTE and VLU in the MVMR analysis nullifies the protective effect of CVI on heart failure, which is consistent with previous research. Our analysis suggests that in the early stages, the symptoms of heart failure may be delayed if combined with CVI, potentially causing clinical confusion. Given the mutual exacerbation between VTE, VLU, and heart failure, closer monitoring plans should be designed for patients with both CVI and heart failure to prevent sudden and drastic deterioration, which may involve more proactive follow-ups, compression therapy, and precautions.

### 4.2 CVI and Atrial Fibrillation

A recent population-based study in Taiwan has attracted attention since it revealed a significant association between varicose veins and a higher risk of atrial fibrillation after adjusting for all confounding variables [16]. While initially challenging to explain, the relationship between veins and atrial fibrillation can be explored. The most common trigger of atrial fibrillation is abnormal action potentials generated by pulmonary vein pathology at the left atrium, a finding that has significantly advanced treatment modalities for atrial fibrillation, such as pulmonary vein isolation [17]. Although the specific mechanisms through which pulmonary vein lesions lead to atrial fibrillation are unclear, Yu-Ki Iwasaki and colleagues demonstrated in animal experiments that fibrosis of the pulmonary veins can induce atrial fibrillation, with detected expression of platelet-derived growth factor (PDGF)-C and vascular endothelial growth factor (VEGF) in fibrotic pulmonary veins [18]. Similarly, increased proliferation of smooth muscle cells and elevated VEGF expression have been observed in CVI [19, 20, 21]. Additionally, the matrix metallopeptidase (MMP) family plays a crucial role in the occurrence of CVI, while high expression of MMPs in atrial tissue has been implicated in the atrial remodeling process [22, 23]. However, whether there are changes in MMP expression in pulmonary veins in the context of atrial fibrillation remains unclear, despite animal experiments suggesting increased MMP-2 in the pulmonary veins of rats with an atrial fibrillation model. Our results also support the claim that there is a correlation between CVI and atrial fibrillation. However, the specific mechanisms still require further exploration.

Some researchers have proposed a theoretical framework suggesting that diseases with common venous system dilating morphological features should be collectively termed “dilating venous disease” [24]. This concept manifests in different vascular territories with distinct clinical presentations. The authors advocate for a systematic assessment of the involvement of other vascular regions in both the arterial and venous systems in patients with any detected dilating disease [24]. If venous dilating diseases, similar to atherosclerosis, involve the entire venous system, lesions in specific areas can promote different pathological manifestations. Combining these previous studies, understanding the connection between atrial fibrillation and lesions of the pulmonary veins through dilating venous diseases can provide insights into the linkage between these two conditions.

### 4.3 CVI and Other Cardiovascular Diseases

We frequently investigate the impact of high arterial pressure on arteries and high venous pressure on veins; however, issues related to the vascular wall itself are frequently overlooked due to the challenges of experimental verification. The abundance of GWAS data now provides an opportunity to investigate the relationships between diseases at a more fundamental level. If there are intrinsic issues with the vascular wall, the occurrence of high arterial or venous pressure in the presence of external factors such as a high sodium–potassium diet or smoking could lead to persistent damage to the vascular wall, causing a vicious cycle. However, aortic aneurysms were also analyzed in our study, and no significant differences were observed. Unlike atrial fibrillation, where the atria are connected to the venous system, aortic aneurysms belong to the arterial system. Whether the observed differences are due to distinct tissue origins remains unknown. We also explored the potentially possible causal effect of CVI on coronary artery atherosclerosis. The great saphenous vein involved in CVI is commonly used as a graft material in coronary artery bypass grafting, and variations or dilations in the great saphenous vein are important factors influencing the choice of graft vessels [25]. Fortunately, our study did not identify a relationship between CVI and coronary artery disease.

### 4.4 Limitations

In this study, we systematically investigated potential causal effects between CVI and CVDs using MR analysis. We employed rigorous methods to select instrumental variables, address pleiotropy concerns, and enhance the validity of the MR analysis. Additionally, we utilized different methods to obtain similar results, further reinforcing the credibility of our findings. However, our study has some limitations. Firstly, the predominant representation of individuals with European ancestry in the GWAS statistics raises concerns regarding the generalizability of our findings to other racial or ethnic populations. It is worth noting that MR is a powerful tool for studying differences in cardiovascular disease risk factors across different ethnic groups [2]. Additionally, while we tried to mitigate pleiotropy, it is improbable that all instances will be completely eliminated in Mendelian randomization studies. Thirdly, subgroup analysis is warranted to investigate further and better understand the impact of CVI on CVDs, such as different age and gender groups, especially different Clinical-Etiology-Anatomy-Pathophysiology (CEAP) classifications of CVI groups.

## 5. Conclusions

This MR study supports the hypothesis that CVI may be implicated in developing CVDs. Our MR estimates provide evidence for the causal effects of genetic liability for CVI on a decreased risk of heart failure and an increased risk of atrial fibrillation. These findings may provide indications for clinicians to enhance the examination and monitoring of CVI during the treatment of CVDs.

## Availability of Data and Materials

The datasets analyzed during the current study are available from the corresponding author on reasonable request.
